# Targeted Degradation of Protein Kinase A via a Stapled Peptide PROTAC

**DOI:** 10.1021/acschembio.4c00237

**Published:** 2024-08-13

**Authors:** Matthew K. Whittaker, George N. Bendzunas, Mahsa Shirani, Timothy J. LeClair, Bassem Shebl, Taylor C. Dill, Philip Coffino, Sanford M. Simon, Eileen J. Kennedy

**Affiliations:** † Department of Pharmaceutical and Biomedical Sciences, College of Pharmacy, University of Georgia, Athens, Georgia 30602, United States; ‡ Laboratory of Cellular Biophysics, The Rockefeller University, New York, New York 10065, United States

## Abstract

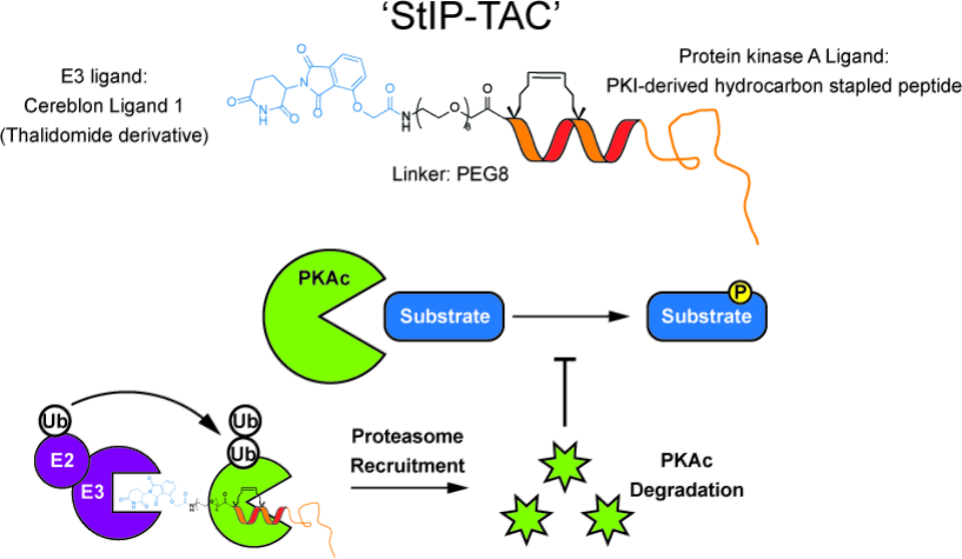

Proteolysis-targeting chimeras (PROTACs) are bifunctional molecules that bind and recruit an E3 ubiquitin ligase to a targeted protein of interest, often through the utilization of a small molecule inhibitor. To expand the possible range of kinase targets that can be degraded by PROTACs, we sought to develop a PROTAC utilizing a hydrocarbon-stapled peptide as the targeting agent to bind the surface of a target protein of interest. In this study, we describe the development of a proteolysis-targeting chimera, dubbed Stapled Inhibitor Peptide - PROTAC or StIP-TAC, linking a hydrocarbon-stapled peptide with an E3 ligase ligand for targeted degradation of Protein Kinase A (PKA). This StIP-TAC molecule stimulated E3-mediated protein degradation of PKA, and this effect could be reversed by the addition of the proteasomal inhibitor MG-132. Further, StIP-TAC treatment led to a significant reduction in PKA substrate phosphorylation. Since many protein targets of interest lack structural features that make them amenable to small molecule targeting, development of StIP-TACs may broaden the potential range of protein targets using a PROTAC-mediated proteasomal degradation approach.

## Introduction

An emerging strategy to inhibit protein function is the use of proteolysis-targeting chimeras, or PROTACs.^1^ PROTACs are bifunctional molecules that join an E3 ligase ligand to a molecule that selectively binds a protein of interest (POI). The POI ligand allows for recruitment of E3 ligases to target proteins, initiating ubiquitination and subsequent proteolysis of the target protein. Advantages of PROTACs include direct target protein degradation, which is not achieved with conventional inhibitors, as well as a catalytic mode of degradation that allows for substoichiometric inhibition of protein targets.^2^ However, current PROTACs largely rely on small molecules as the POI ligand, which limits their applicability to “undruggable” protein targets lacking well-characterized ligands or structural pockets required for ligand binding.^3^

Recent studies have explored the use of peptides as the POI ligand to improve target selectivity of PROTACs while retaining high binding affinity.^4−8^ Peptides present an advantage in their ability to bind conventionally “undruggable” proteins lacking small pockets, however, unmodified peptides are limited by a lack of proteolytic stability and poor cell penetrance.^9^ As such, current peptide-based PROTACs are also limited by the poor stability and poor cellular penetration.^3^ One method to improve peptide stability and thereby effectivity is via all-hydrocarbon “stapling” by incorporating α,α-disubstituted olefinic amino acids into the peptide sequence followed by ring-closing metathesis to form an olefin brace that constrains the peptide into an α-helix.^10^ All-hydrocarbon stapling can enhance α-helicity, cellular penetrance, and proteolytic stability of peptides compared to unstapled counterparts.^11,12^ Via introduction of a staple, several groups have demonstrated improved stability and cellular penetrance of peptide-based PROTACs for various protein targets including β-catenin,^13^ estrogen receptor α,^14^ and murine double minute 2 (MDM2)/murine double minute X (MDMX).^15^ Applying this strategy, the development of peptide-based PROTACs targeting kinases may serve as an alternative approach for targeted kinase inhibition.

The kinase superfamily is one of the largest classes in the human genome with over 500 members and comprises the second-most targeted class of proteins in therapeutic development.^16^ By degrading rather than simply inhibiting targeted proteins, PROTACs could help to overcome resistance mechanisms associated with conventional kinase inhibitors, such as protein overexpression or point mutations, while also targeting kinase scaffolding functions integral to disease states.^17^ A current limitation of kinase-targeting PROTACs is reliance on small molecule kinase inhibitors as the POI ligand, which could result in promiscuous binding of nontargeted kinases as well as reduced targetability of kinases lacking well-characterized and specific inhibitors.^17−20^ Although hydrocarbon stapling has been applied to develop peptide inhibitors for a variety of kinase targets including PKA,^21,22^ EGFR,^23^ and PKC,^24^ there currently exist no PROTACs exploiting the selective allosteric binding of stapled peptides to target a kinase for degradation. The compound described in this study utilizes the emerging strategy of incorporating an all-hydrocarbon stapled peptide as the POI ligand as a strategy for kinase targeting.

As a model system, Protein Kinase A (PKA) is a prototypical kinase and one of the best-studied kinases to date. Aberrant activation of PKA is implicated in a variety of diseases including cancer and metabolic and endocrine disorders.^25^ Endogenous inhibition of PKA activity was discovered with the pseudosubstrate inhibitor peptide, PKI.^26^ PKI is regarded as being a selective kinase inhibitor of PKA as it binds to a unique pseudosubstrate pocket on PKA that, although shared by other members of the AGC kinase family, has unique structural features on PKA that bestow considerably higher affinity and selectivity for PKI over all other AGC kinases.^27−30^ Nonspecific kinase inhibition by PKI was previously reported, primarily CAMK1^29^ and PKG,^31^ however, inhibition required PKI concentrations that were considerably higher than required for PKA inhibition. The primary small molecule inhibitor used for targeting PKA, H89, is substantially more promiscuous and has been identified to inhibit a variety of other kinases with inhibition of MSK1, S6K1 and ROCK-II occurring with similar or greater potency than that of PKA.^32^ In addition, H89 was also found to inhibit other nonkinase targets including ion channels, RhoA, and Ca^2+^-ATPase.^33^ Based on these prior observations of notable kinase selectivity, we reasoned that incorporating a PKI-derived peptide as the POI ligand may serve as an alternative PKA-targeting molecule and starting point for developing a stapled PROTAC.

Building upon this allosteric inhibitor for PKA, we sought to explore the possibility of allosterically targeting a kinase for degradation via the development of a stapled peptide PROTAC. It was previously shown that a linear sequence comprised of the first 24 residues of PKI contains most residues essential for binding to and inhibiting the kinase activity of the PKA catalytic subunit.^28^ Since native PKI is not membrane permeable, a modified all-hydrocarbon stapled peptide analog of PKI_1–24_ was previously developed that was demonstrated to bind PKA with a subnanomolar affinity and could permeate cells and inhibit intracellular PKA activity.^21^ Here, we report the development of an all-hydrocarbon stapled peptide-based PROTAC molecule derived from PKI that is linked to a cereblon ligand. This stapled PROTAC, termed StIP-TAC, was found to bind PKA and promote PKA degradation in a proteasomal-dependent fashion. Further, this loss of protein was also found to correlate with loss of PKA activity in cells, thereby demonstrating targeted downregulation of kinase signaling.

## Results and Discussion

**Figure 1:**
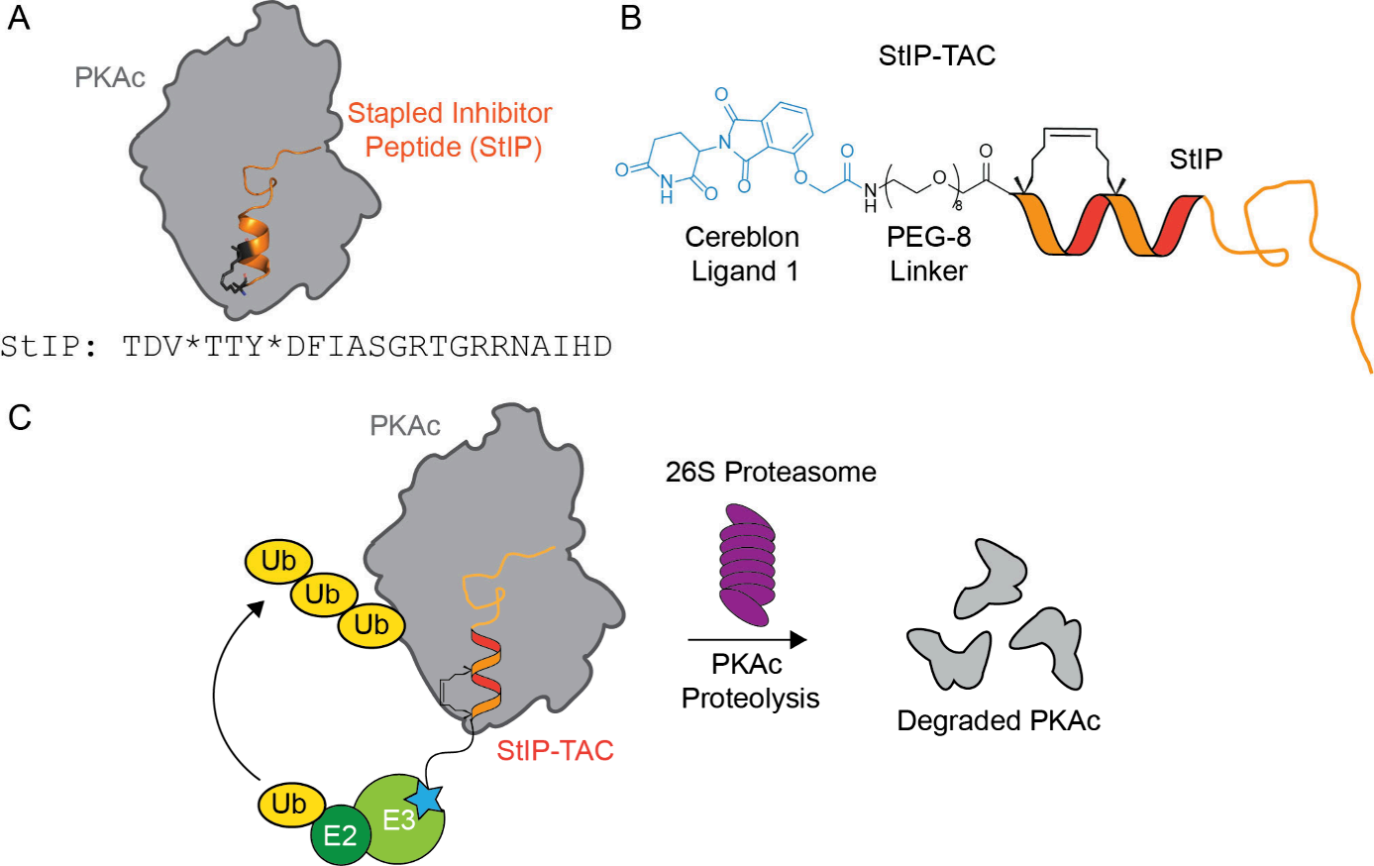
**Schematic of PROTAC design and activity.** (A) Schematic of StIP binding to PKA along with parent sequence. * = (S)-2-(4-pentenyl)alanine. (B) Schematic of PROTAC structure. StIP-derived peptide is conjugated to thalidomide-derived Cereblon Ligand 1 via a PEG-8 linker. (C) Proposed mechanism of action of PKI-derived PROTAC molecule. StIP-derived POI ligand binds PKA and recruits E3 ligase complex via Cereblon Ligand 1, resulting in ubiquitination (Ub) of PKA and subsequent targeted degradation by the proteasome. E2 = E2 ligase, E3 = E3 ligase.

**Figure 2:**
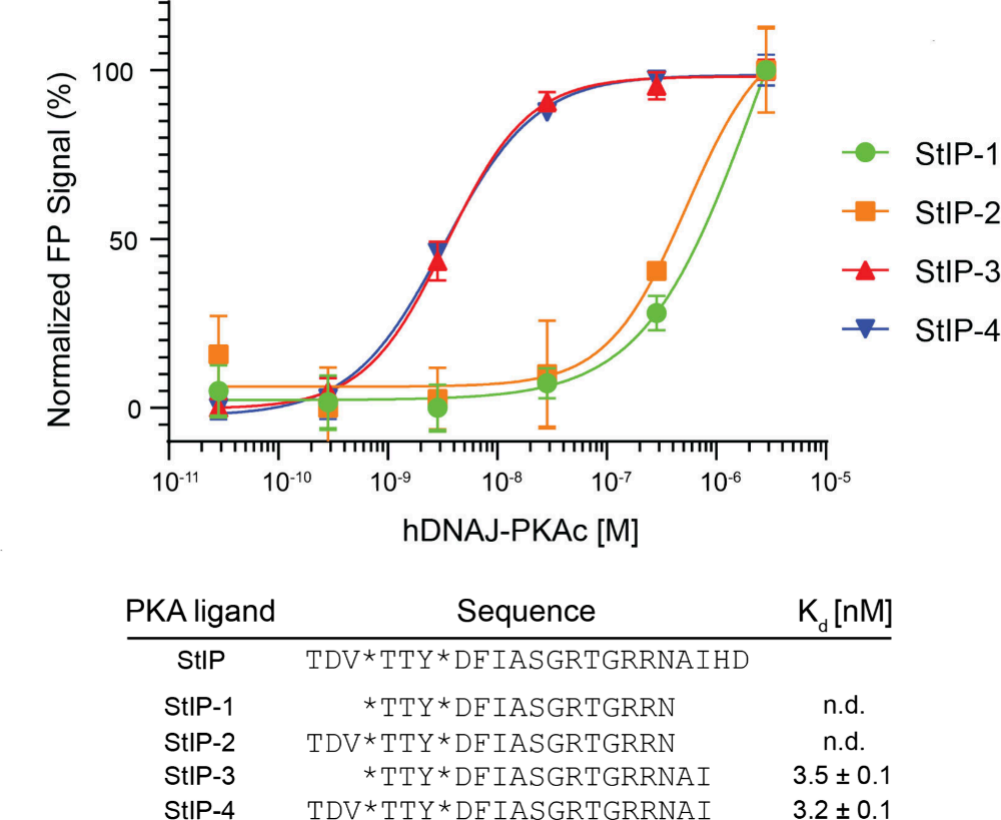
**Direct binding measurements of fluorescein-labeled truncated peptides to PKA catalytic subunit determined by fluorescence polarization.** StIP-3 and StIP-4 demonstrate low nanomolar binding to PKA with truncated sequences compared to the parent peptide sequence, StIP. * = location where (S)-2-(4-pentenyl)alanine is incorporated into the peptide sequence to form the hydrocarbon staple. Mean values and standard deviation of three independent measurements are given. n.d. = not determined. Both StIP-T3 and StIP-T4 were found to have low-nanomolar affinities.

As kinase-targeting PROTACs rely primarily on small molecule kinase inhibitors as the POI ligand, we sought to develop an allosteric inhibitor of PKA activity using the emerging strategy of stapled peptide PROTACs. Using PKA as a model system, we aimed to conjugate a stapled peptide mimic of the endogenous pseudosubstrate inhibitor PKI to a thalidomide-derived E3 ligase ligand, Cereblon ligand 1, to allow for ternary complex formation and recruitment of the proteasome to PKA (Figure 1). We began by designing a library of truncated, PKI-derived stapled peptide compounds to serve as the POI ligand and achieve PKA binding. Our lab previously reported a stapled-peptide mimic of the PKA pseudosubstrate inhibitor PKI that achieved picomolar binding to the catalytic subunit of PKA, effectively permeated cells, and inhibited PKA substrate phosphorylation in the low micromolar range.^21^ Based on this PKI-derived sequence, we developed a library of truncated peptides to identify a shortened POI ligand that retained parental binding while reducing the overall size of our PKA-targeting PROTAC molecule (Figure 2).

To determine the binding affinity of the truncated library toward the catalytic subunit of PKA, fluorescence polarization (FP) experiments were performed using N-terminally fluorescein-labeled PKI-derived peptides incubated with a range of concentrations of recombinant human DNAJB1-PKAc (Figure 2). The DNAJB1-PKAc chimera is a clinically relevant protein identified in fibrolamellar hepatocellular carcinoma (FL-HCC) patients that combines the chaperonin-binding domain of heat-shock protein 40 (HSP40/DNAJ) with the catalytic (c) subunit of PKA.^34^ The active site of the DNAJB1-PKAc protein is identical to native PKAc and retains inhibition by PKI with a nearly identical binding conformation.^35^ In addition, the fusion protein retains comparable enzyme kinetics and binding affinity to PKI and PKI-derived peptides as compared to wild-type PKAc.^36^ Based on this screen, two stapled peptides, StIP-3 and StIP-4, were found to have a low-nanomolar binding affinity (3–4 nM) (Figure 2). From this data, we chose StIP-3 as our lead POI ligand, as it possessed low-nanomolar binding affinity to PKAc comparable to StIP-4 but had a slightly shorter sequence, thereby reducing the overall molecular weight of the peptide and resulting PROTAC. To generate the StIP-TAC, several PEG linker lengths were conjugated with the thalidomide-derived Cereblon Ligand 1 and preliminarily tested (PEG_3_ to PEG_9_; Figure S2). All PEG lengths appeared to yield compound activity, and thus the PEG_8_ linker was chosen to provide greater flexibility to the overall compound.

**Figure 3:**
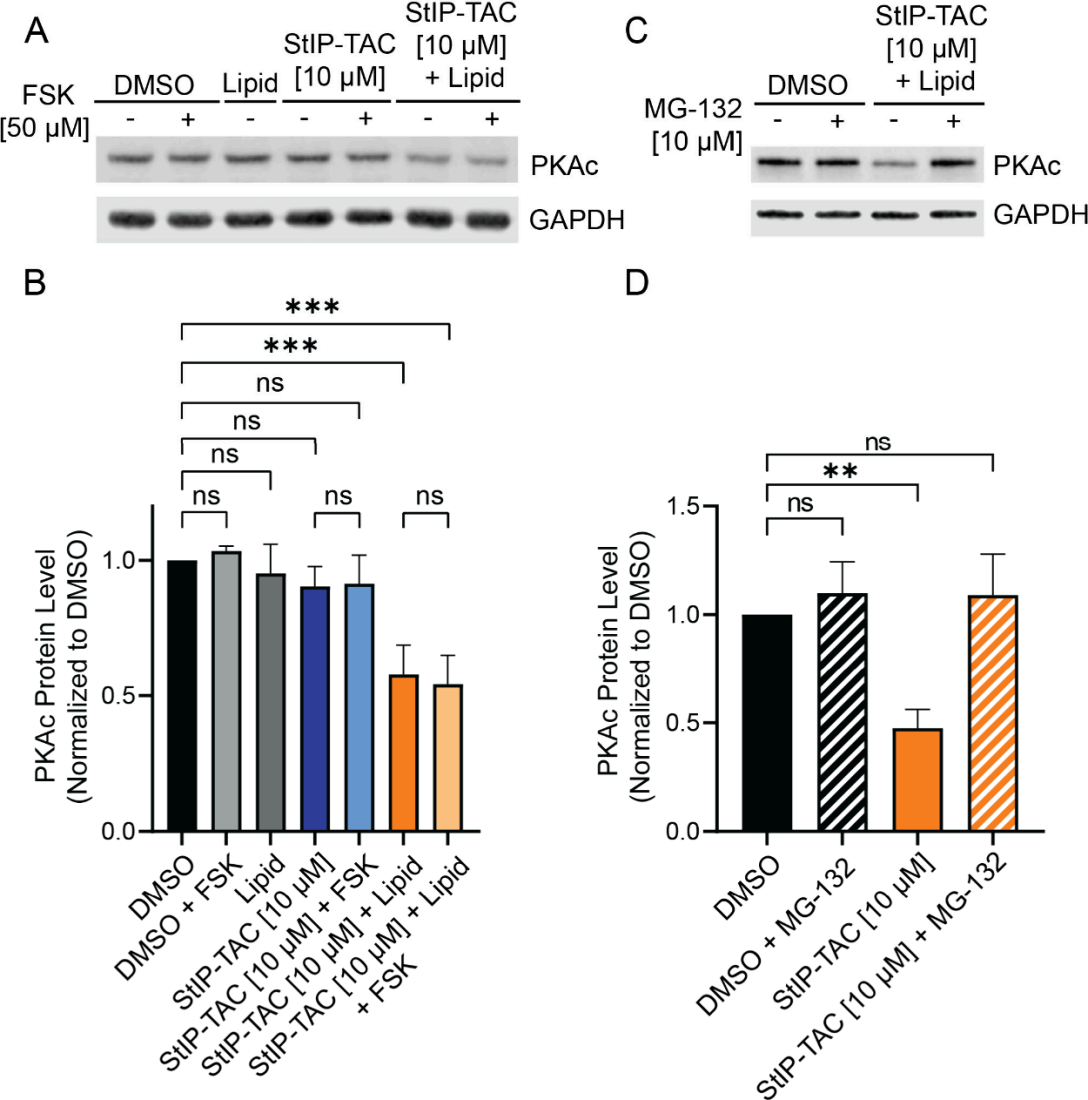
**StIP-TAC treatment induces PKA degradation.** (A) Representative Western blot of cells treated with vehicle control, StIP-TAC, and StIP-TAC + SAINT Protein reagent (Lipid) for 5 h (*n* = 3) with or without a 30 min forskolin (FSK) stimulation. Cells treated with StIP-TAC in conjunction with the SAINT Protein lipid delivery formulation demonstrate reduced PKA protein levels. (B) Densitometric quantification of three independent Western blots (*n* = 3) demonstrates a statistically significant reduction in PKA protein levels for cells treated with StIP-TAC and SAINT Protein reagent. No significant difference in PKA levels was detected between forskolin-stimulated and unstimulated treatments. Quantification was performed via Li-COR Image Studio. PKA bands for each treatment were normalized to the GAPDH loading control and compared to the DMSO control treatment. ***p < 0.001; ns, not significant as assessed by one-way ANOVA and Bonferroni’s multiple comparisons test. Error bars represent standard deviation. (C) Representative Western blot of cells treated with 10 μM proteasomal inhibitor MG-132 with vehicle control or StIP-TAC with SAINT protein reagent for 5 h (*n* = 3). Cells treated with StIP-TAC and SAINT protein demonstrate a reduction in PKA protein levels that are rescued with the addition of proteasomal inhibitor. (D) Densitometric quantification of three independent Western blots (*n* = 3) demonstrating a statistically significant reduction in PKA protein levels in cells treated with StIP-TAC and SAINT Protein reagent that is rescued to near-baseline levels upon proteasomal inhibition. Quantification was performed in Li-COR Image Studio. PKA bands for each treatment were normalized to the GAPDH loading control and compared to the DMSO control treatment. **p = 0.003; ns, not significant as assessed by one-way ANOVA and Bonferroni’s multiple comparisons test.

To determine whether StIP-TAC could successfully degrade native PKAc in cells, MDA-MB-231 cells were used. Initial screening revealed a slight, albeit insignificant reduction in PKAc protein levels (Figure 3A,B). We hypothesized this lack of a statistically significant effect may be a result of the compound’s relatively large molecular weight (∼3 kDa) and potentially limited cellular permeability, a common limitation of PROTAC molecules.^37−40^ A cationic lipid-based transfection reagent, SAINT Protein (Synvolux), was demonstrated to improve cellular uptake and activity of other hydrocarbon stapled peptides via more efficient release of peptides into the cytoplasm through vesicular escape.^41^ Based on this study, we aimed to improve delivery of our compound using SAINT Protein. When cells were treated for 5 h with 10 μM StIP-TAC, no significant reduction in PKAc protein levels was observed, however, treatment of cells with 10 μM StIP-TAC alongside SAINT Protein lipid reagent led to a significant reduction of PKAc protein levels by approximately 50% as compared to the vehicle control (Figure 3A,B; Figure S3). As activation of PKA by cAMP is a requisite for PKI binding, we assessed whether forskolin stimulation would impact StIP-TAC activity, however, no significant difference was observed in PKAc degradation between forskolin stimulated and unstimulated controls (Figure 3A,B). Confirming that SAINT-Protein lipid reagent did not impact PKAc protein levels, we observed no significant reduction in PKAc protein levels in cells treated with SAINT Protein reagent alone (Figure 3B; Figure S4). To confirm that StIP-TAC-mediated degradation of PKAc resulted from recruitment of the proteasome, we incubated cells with 10 μM StIP-TAC alongside 10 μM proteasomal inhibitor MG-132 and subsequently screened for PKAc protein levels. Co-treatment of StIP-TAC-treated cells with MG-132 rescued PKAc protein to baseline levels, thereby demonstrating that the reduction in PKAc was dependent on proteasomal degradation (Figure 3C,D; Figure S5).

Cereblon is a member of the cullin RING ligase (CRL) family, which requires initial activation by conjugation of NEDD8 protein to the cullin protein via NEDD activating enzyme (NAE) before ubiquitination of target proteins may occur. To confirm that the StIP-TAC activity resulted from CRL activation and recruitment, we incubated cells with 10 μM StIP-TAC alongside 6 μM of the NAE inhibitor MLN4924 and subsequently screened for PKAc protein levels. Co-treatment of StIP-TAC treated cells with MLN4924 rescued PKAc protein to baseline levels and demonstrated that StIP-TAC degradation of PKAc was mediated via NAE activation of the CRL (Figure S6). Additionally, to confirm that the activity of StIP-TAC resulted from the functionality of the Cereblon ligand, we incubated cells with equimolar StIP-3, which retains the identical PKAc binding portion of StIP-TAC but lacks the Cereblon ligand, with or without SAINT Protein lipid reagent. Treatment of cells with StIP-3, regardless of the presence of SAINT Protein, led to no significant reduction in PKAc levels relative to DMSO, demonstrating that the functionality of StIP-TAC depends on the presence of the thalidomide-derived Cereblon ligand (Figure S6).

**Figure 4:**
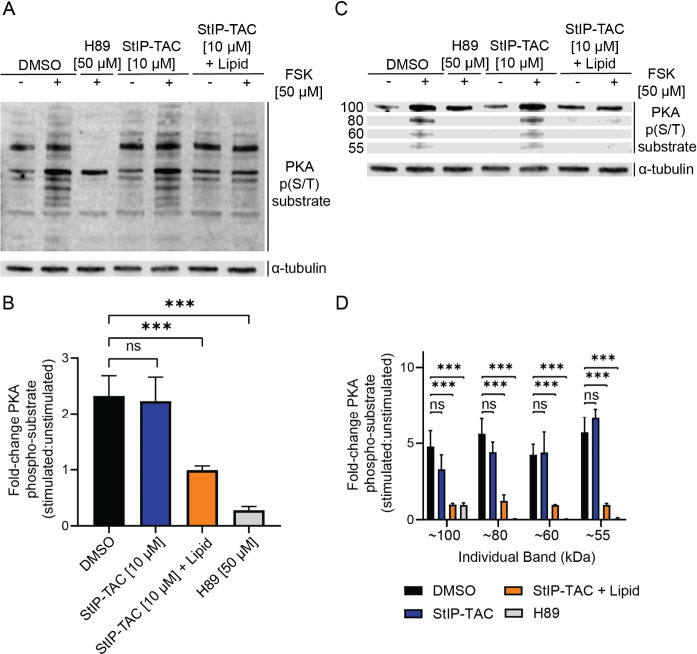
**StIP-TAC treatment inhibits phosphorylation of PKA substrates.** (A) Representative Western blot of cells treated with vehicle control, H89, StIP-TAC, or StIP-TAC + SAINT Protein reagent for 5 h with or without 30 min stimulation with 50 μM forskolin (*n* = 3). Cells treated with H89 and StIP-TAC alongside SAINT protein display a reduction in fold change of total phosphorylated PKA substrate levels after forskolin stimulation. (B) Densitometric quantification of Western blots for three independent experiments (*n* = 3) demonstrating a statistically significant reduction in total PKA substrate phosphorylation in cells treated with H89 and StIP-TAC with SAINT Protein reagent. Quantification was performed via Li-COR Image Studio. *** *p* < 0.001; ns, not significant as assessed by one-way ANOVA and Bonferroni’s multiple comparisons test. Error bars represent standard deviation. (C) Representative Western blot image of select PKA phosphorylated substrates at approximately 55, 60, 80, and 100 kDa bands, demonstrating a prominent reduction in phosphorylation after forskolin stimulation upon treatment with StIP-TAC and SAINT Protein reagent (*n* = 3). (D) Densitometric quantification of Western blots from three separate experiments (*n* = 3), demonstrating a statistically significant reduction in phosphorylation of the approximately 55, 60, 80, and 100 kDa substrate bands for cells treated with StIP-TAC and SAINT Protein reagent. Quantification was performed via Li-COR Image Studio. ***p < 0.001; ns, not significant as assessed by one-way ANOVA and Bonferroni’s multiple comparisons test. Error bars represent standard deviation.

Next, we sought to assess whether StIP-TAC could also downregulate PKAc signaling in cells. For this experiment, cells were treated with forskolin to stimulate PKAc activity. Cells were either treated with DMSO, the small molecule PKAc inhibitor H89, StIP-TAC alone, or StIP-TAC with SAINT protein (Figure 4; Figure S7). Similar to the observed reduction in PKAc levels, treatment of cells with StIP-TAC alone did not yield a significant reduction in PKAc substrate phosphorylation, however, cells treated with StIP-TAC along with SAINT Protein led to a significant reduction in substrate phosphorylation to near basal levels despite stimulation with forskolin (Figure 4A,B). Interestingly, the most prominent change in substrate phosphorylation was consistently observed for particular substrates that migrated as approximately 55, 60, 80, and 100 kDa bands. (Figure 4C,D). A known PKA substrate, vasodilator-stimulated phosphoprotein (VASP),^42^ was also probed for PKA-mediated phosphorylation (Figure S8). Similar results were noted where treatment of cells with vehicle or StIP-TAC alone did not result in a significant reduction in VASP phosphorylation in the presence of forskolin, however, treatment of cells with StIP-TAC along with SAINT-Protein led to a significant inhibition of VASP phosphorylation, thereby further confirming inhibited PKA substrate phosphorylation.

The novel compound presented in this study, StIP-TAC, achieved significant reduction in protein levels for PKA as well as inhibition of PKA substrate phosphorylation when combined with a commercially available lipid carrier, SAINT protein. StIP-TAC-induced PKA degradation was found to be proteasome-dependent, as cotreatment with the proteasomal inhibitor MG-132 as well as the NAE inhibitor MLN4924 led to a rescue of PKA protein to baseline levels. Building from our previous work,^21^ we identified a suitable POI ligand via developing and screening a library of truncated stapled peptide mimics of the endogenous PKI inhibitor peptide. This work demonstrates a novel strategy to target PKA. Interestingly, in several studies screening the kinome for PROTAC-mediated degradability using PROTACs incorporating a promiscuous small molecule kinase inhibitor as the POI ligand, PKAc was not identified as a degradable target.^18−20^ The work presented in this study may thereby highlight the utility of incorporating allosteric stapled peptide inhibitors, such as all-hydrocarbon stapled peptides, as the POI ligand to expand the targetability of kinases and other proteins not amenable to small molecule binding. Future studies will need to be undertaken to determine the generalizability of this strategy toward the kinome superfamily as well as how this compound may compare to other kinase degraders.

Although hydrocarbon staples can improve cellular permeability of peptides, designing peptides to improve cell permeability still remains a challenge and is dependent on a variety of factors such as overall hydrophobicity, charge, and staple placement.^43−45^ While the addition of the hydrocarbon staple does not guarantee improved cell permeability,^46^ incorporation of the hydrocarbon staple has been widely shown to improve the stability of peptides^10,12,47−50^ and peptide-PROTACs,^5,15,51,52^ and thus may retain value for improving the overall properties for a peptide of interest. The stapled mimic of PKI developed in our lab that served as the template for StIP-TAC was able to overcome the lack of cell permeability associated with native PKI without the need for conventional modifications such as myristoylation.^21^ In this study, we found that once the PEG and cereblon moieties were added to the peptide, the compound was no longer permeable. Incorporation of these additional groups alters the overall physicochemical properties of the StIP compound, resulting in impacted intrinsic permeability of the StIP-TAC. Therefore, we chose to incorporate the SAINT-protein reagent based on a prior study where SAINT-PhD combined with their peptide (the precursor to SAINT-protein) led to enhanced peptide uptake in order to identify active compounds that were initially hindered by poor permeability.^41^ However, overcoming the permeability barrier via the usage of SAINT-Protein allowed for the observation of activity for StIP-TAC in cells. This raises the important point that although hydrocarbon peptide stapling can enable cell permeation, permeation has its limits that may be dictated by a variety of factors including the overall molecular weight.

Building from conventional inhibitors, the emerging strategy of PROTACs allows not only for inhibition but direct catalytic degradation of protein targets. In doing so, PROTACs can circumvent issues with conventional inhibitors including drug resistance and a compensatory increase in protein levels while maintaining substoichiometric inhibitory activity.^38^ The POI ligand of most reported PROTACs consists of a small molecule, limiting the selectivity of the PROTAC as a whole.^3^ However, the incorporation of stapled peptide ligands combining the improved selectivity of peptides with the improved stability and cellular penetrance conferred by the staple may prove a useful strategy to improve the overall activity of PROTACs. Stapled peptide PROTACs may serve as a useful strategy to broaden the scope of targetable POIs for proteasomal-mediated degradation.

As hydrocarbon stapled peptides and their resulting peptide PROTACs can be synthesized with relative ease, the stapled peptide-based framework for PROTAC development presented in this study and others may prove a novel strategy for developing selective degraders of target proteins. One limitation of stapled peptide PROTACs is their relatively large molecular weight that can limit their permeability and resultingly, activity.^3^ However, by using delivery systems such as the SAINT Protein reagent outlined in this study, it may be possible to improve cellular penetrance and activity of these compounds. As the large molecular weight of many PROTACs resultingly hinders compound uptake and activity, strategies additional to SAINT-Protein to package peptide and proteins for systemic delivery - including liposome or lipid nanoparticle formulations - could potentially be applied to efficiently deliver molecules like StIP-TAC *in vivo*.^53^ Furthermore, the ability to modulate PROTAC structure via truncation of the POI ligand, selection of alternate linkers and E3 ligands, and incorporation of cell-penetrating peptide sequences could allow for the screening of compounds with reduced size and improved permeability. In addition to PKI, synthetic peptide inhibitors derived from naturally encoded pseudosubstrate sequences have been developed to target kinases including PKG, PKC, and GSK-3.^54^ Using the strategy outlined in this study, it is possible that such inhibitor peptide sequences could be developed into stapled peptide PROTACs to allow for the selective degradation of additional kinase targets.

In addition, future studies will be needed to compare the metabolic stability of these relatively large PROTAC compounds. Incorporation of the all-hydrocarbon staple into various sequences was found to improve the metabolic stability of peptides,^55^ presumably by confining the amide backbone in the helical core, thereby protecting the sequence from proteolysis. Additionally, incorporation of the hydrocarbon staple has been demonstrated to improve the stability and activity of peptide-based PROTACs compared to their unmodified counterparts in several studies.^5,15,51,52^ Thus, although improved metabolic stability is expected, the molecule half-life and its relative comparison to small molecule-based PROTACs will need to be assessed.

Clinically, the DNAJ-PKA fusion protein has been found in almost all FL-HCC patients,^34^ and is sufficient to trigger FL-HCC.^56,57^ Elimination of the transcript for DNAJB1::PRKACA is sufficient to kill the tumor.^58^ Conventional kinase inhibition of the DNAJ-PKA chimera is challenged by the DNAJ promoter leading to three- to 8-fold elevation of chimeric protein levels relative to native PKA,^34^ however, the catalytic mode of PROTACs that allows for substoichiometric inhibition could help to circumvent this oncogenic overexpression. It is possible that by developing a stapled peptide ligand that selectively binds the oncogenic DNAJ-PKA protein or by localizing StIP-TAC to FL-HCC cells, a modified StIP-TAC could be developed to selectively target the oncogenic fusion protein while maintaining intrinsic kinase activity in noncancerous cells.

## Supporting Information Available

Table S1: Compound sequences and masses determined via HPLC-MS, Figure S1: LCMS Spectra for StIP-TAC, Figure S2: Degradation and phosphorylation control experiments for DMSO and SAINT-Protein lipid alone, Figure S3: Supplemental Western blots for PKA degradation experiment, Figure S4: Supplemental Western blots demonstrating that SAINT-Protein does not affect PKAc levels or substrate phosphorylation, Figure S5: Supplemental Western blots MG-132 rescue experiment, Figure S6: Supplemental Western blots demonstrating that StIP-TAC activity is dependent on the neddylation pathway, Figure S7: Supplemental Western blots for PKA substrate phosphorylation experiment, Figure S8: StIP-TAC inhibits phosphorylation of the PKA substrate VASP (PDF)
