# Supplementary material for: Targeted Degradation of Protein Kinase A via a Stapled Peptide PROTAC
Source: ACS Chem Biol. 2024 Aug 13;19(9):1888–95. doi: 10.1021/acschembio.4c00237 (PMC11420944; doi:10.1021/acschembio.4c00237)
Supplement: Supplementary file 1 — cb4c00237_si_001.pdf [file cb4c00237_si_001.pdf]

Targeted degradation of Protein Kinase A via a stapled peptide PROTAC

Matthew K. Whittaker[1], George N. Bendzunas[1], Mahsa Shirani[2], Timothy J. LeClair[1],

Bassem Shebl[2], Taylor C. Dill[1], Philip Coffino[2], Sanford M. Simon[2], Eileen J. Kennedy[1]

[1]Department of Pharmaceutical and Biomedical Sciences, College of Pharmacy, University of Georgia, Athens, Georgia 30602, United States
[2]Laboratory of Cellular Biophysics, The Rockefeller University, New York, New York 10065, United States

* Corresponding author: Eileen Kennedy (ekennedy@uga.edu)

MATERIALS AND METHODS

Peptide and PROTAC synthesis. Rink amide MBHA resin, N-α-Fmoc protected amino acids were purchased from Novabiochem (Merck Millipore). (S)-N-Fmoc-2-(4'-pentenyl)alanine and Grubb's catalyst (1st generation) were purchased from Sigma-Aldrich. Fmoc-NH-PEG_8-CH2COOH was purchased from ChemPep Inc. and 2-[[2-(2,6-dioxo-3-piperidinyl)-2,3-dihydro-1,3-dioxo-1H-isoindol-4-yl]oxy]-acetic acid (Cereblon Ligand 1) was purchased from Cayman Chemicals.

Peptides were synthesized via standard Fmoc (fluorenylmethoxycarbonyl) protected solid phase peptide synthesis. Rink amide MBHA resin was equilibrated in NMP (1-methyl-2-pyrrolidinone). Fmoc protecting group was removed using a 25% v/v solution of piperidine in NMP for 25 minutes and followed by three washes with NMP. Standard amino acid coupling was performed with 10 eq. Fmoc-protected amino acid followed with the addition of 9.9 eq. HCTU [O-(1H-6-Chlorobenzotriazole-1-yl)-1,1,3,3,-tetramethyluronium hexafluorophosphate] and 20 eq. DIEA (N, N — Diisopropylethylamine). Coupling with of amino acid was performed for at least 45 minutes prior to three washes in NMP. Coupling with Fmoc-S_5 [(S)-2-(4-pentenyl)alanine], Fmoc-NH-PEG_3-CH_2COOH and Fmoc-NH-PEG_8-CH_2COOH were performed using 4 eq. for 45 minutes followed with repeated addition of 10 eq. DIEA and gentle agitation for an additional 45 minutes. After the addition of the last S_5 residue and before the addition of PEG, olefinic ring-closing metathesis was performed on Fmoc-protected, resin-bound peptide with 0.4 equivalents of Grubb's first-generation catalyst [Benzylidene-bis(tricyclohexylphosphine)dichloro ruthenium] in DCE (1,2-dichloroethane) for 1 hour and repeated once to ensure complete formation of the

hydrocarbon staple. For coupling of 5(6)-carboxyfluorescein (FAM), 2 eq. FAM were added alongside 1.8 eq. HCTU and 4.6 q. DIEA in DMF with gentle agitation. After 1 hour of coupling, an additional 4.6 eq. DIEA was added and the reaction was allowed to couple overnight. Coupling of Cereblon Ligand 1 was performed using 2.5 eq. of Cereblon Ligand 1 followed by the addition of 2.475 eq. HCTU and 10 eq. DIEA. After 1 hour of gentle agitation, an additional 10 eq. of DIEA was added and the reaction was allowed to proceed overnight. Resin cleavage was performed in 95% (v/v) triofluoroacetic acid (TFA), 2.5% (v/v) triisopropylsilane and 2.5% (v/v) water for 4-5 hours at room temperature. The cleaved peptide was filtered through glass wool and precipitated in methyl-tert-butyl ether via centrifugation at 2800 rpm for 30 minutes at 4 C. Pelleted crude peptide was allowed to dry overnight under a gentle air stream.

Peptides were characterized using ES-MS (Agilent 6120 Single Quadrupole, Santa Clara, CA, USA) after HPLC separation over a Zorbax analytical SB-C18 column (Agilent 1200). Peptides were separated by reverse phase HPLC over a 10-100% gradient of water:acetonitrile with 0.1% TFA using a linear gradient and flow rate of 0.5 mL/min. Purification was performed via the same conditions, however, using a semi-preparatory Zorbax SB-C18 column with a flow rate of 4.0 mL/min. FAM-labeled peptides were quantified based on absorbance at 495 nm in 10 mM Tris pH 8 using an extinction coefficient of e = 68,000 $M^{-1}cm^{-1}$ as described previously. PROTAC was quantified via a combination of 280 nM absorbance in a microplate reader (Bio-Tek Synergy 2) in 6M Guanidine HCl pH 6.5 as well as confirmation with final dried product mass. Purified peptide masses determined via LC-MS for each FAM-labeled product are as follows: StIP-1 (FAM-PEG-3-*TTY*DFIASGRTGRRN) = 2511.0 (expected mass

= 2511.7); StIP-2 (FAM-PEG3-TDV*TTY*DFIASGRTGRRN) = 2826.9 (expected mass = 2827.1), StIP-3 (FAM-PEG3-*TTY*DFIASGRTGRRNAI) = 2695.2 (expected mass = 2696.0), StIP-4 (FAM-PEG3-TDV*TTY*DFIASGRTGRRNAI) = 3010.5 (expected mass = 3011.3). Peptide mass for StIP-TAC (Cereblon Ligand 1-PEG8-*TTY*DFIASGRTGRRNAI) = 2871.9 (expected mass = 2872.2).

Fluorescence Polarization (FP) Assay. Binding studies for the truncated peptide library to DNAJ-PKA-C were performed according to protocols described previously [21]. FAM-labeled peptides were plated to a final concentration of 0.5 nM after the addition of protein in 384-well microtiter plates. A tenfold dilution series of DNAJ-PKA-C were mixed in peptide wells to have 6 final concentrations ranging from 3 μM to 30 pM. Assays were performed using FP buffer (20 mM MOPS pH 7, 150 mM NaCl, 0.005% CHAPS, 1 mM ATP, 10 mM $MgCl_2$) at room temperature. All data were obtained using a BioTek Synergy 2 microplate reader using the following settings: excitation/emission/dichroic filters: 485-20 nm/528-20 nm/510 nm, 50 flashes per well, settling time 0.1 s. Non-linear regression (sigmoidal dose-response) was performed using GraphPad Prism 6.01 on FP signals plotted against log-scale protein concentrations to determine equilibrium dissociation constants (K_D). Data analysis was performed using GraphPad Prism (Dotmatics).

Cell culture. MDA-MB-231 (HTB-26, ATCC) cells were cultured at low passage number in RPMI medium with 10% FBS and 1% penicillin-streptomycin (Gibco) and grown on tissue culture plates (BD Biosciences) at 37 °C and 5% CO_2.

Cell-based PROTAC activity assays. MDA-MB-231 cells were grown on 6-well tissue culture plates (BD Biosciences). Cells were serum-starved for 24 hours in serum-free RPMI with penicillin/streptomycin, glucose, and L-glutamine. StIP-TAC treatment was prepared with SAINT-Protein reagent according to manufacturer protocol (Synvolux) and added to cells at a final concentration of 10 μM for 5 hours. For phosphorylation studies, cells were additionally stimulated with 50 μM forskolin for 30 minutes before harvesting. For H89-treated cells, 50 μM H89 was added 30 minutes before stimulation with forskolin. For degradation studies, cells were co-treated with 10 μM MG-132 (Selleck Chemicals) alongside StIP-TAC and DMSO treatments for the duration of the 5-hour experiment. For studies examining the NAE-mediated activation of CRL, cells were co-treated with 6 μM MLN4924 (Cayman Chemical) alongside StIP-TAC for the duration of the 5-hour experiment. For studies examining potential effects of StIP-3 on PKAc protein levels, cells were treated with 10 μM StIP-3 with or without SAINT-Protein reagent for 5 hours. Cells were harvested in Laemmli sample buffer, boiled for 10 minutes and protein levels were analyzed via western blotting. For control experiments, cells were incubated for 5 hours with an equivalent volume of DMSO or SAINT-Protein to the above studies, with or without forskolin stimulation.

To assess PKA degradation, anti-PKAc (1:1000, BD Biosciences) and anti-GAPDH (1:1000, Proteintech) primary antibodies were used. To assess PKA substrate phosphorylation, anti-phospho serine/threonine PKA substrate (1:1000, Cell Signaling Technology) and anti-tubulin (1:1000, Cell Signaling Technology) primary antibodies were used. To assess phosphorylation of VASP, anti-phospho VASP (1:1000, Cell

Signaling Technology) and anti-GAPDH (0.5 µg/mL, DSHB) primary antibodies were used. Incubation with primary antibodies was followed by incubation with anti-mouse 680RD (1:15,000) and anti-rabbit 800CW (1:15,000) secondary antibodies (Li-COR Biosciences). Western blot fluorescence detection was performed using a LI-COR Odyssey Fc imaging system. Data analysis was performed using GraphPad Prism (Dotmatics).

Name	Sequence	Expected Mass	Observed Mass
StIP-1 FAM	(5/6 FAM)-PEG3-*TTY*DFIASGRTGRRN	2511.7	2511.0
StIP-2 FAM	(5/6 FAM)-PEG3-TDV*TTY*DFIASGRTGRRN	2827.1	2826.9
StIP-3 FAM	(5/6 FAM)-PEG3-*TTY*DFIASGRTGRRNAI	2696.0	2695.2
StIP-4 FAM	(5/6 FAM)-PEG3-TDV*TTY*DFIASGRTGRRNAI	3011.3	3010.5
StIP-TAC	Cereblon Ligand 1 -PEG8-*TTY*DFIASGRTGRRNAI	2872.2	2871.9

Table S1. Peptide and PROTAC sequences, expected and observed masses determined via HPLC-MS. Asterisks (*) represent residues of (S)-2-(4-pentenyl)-alanine (S_5).

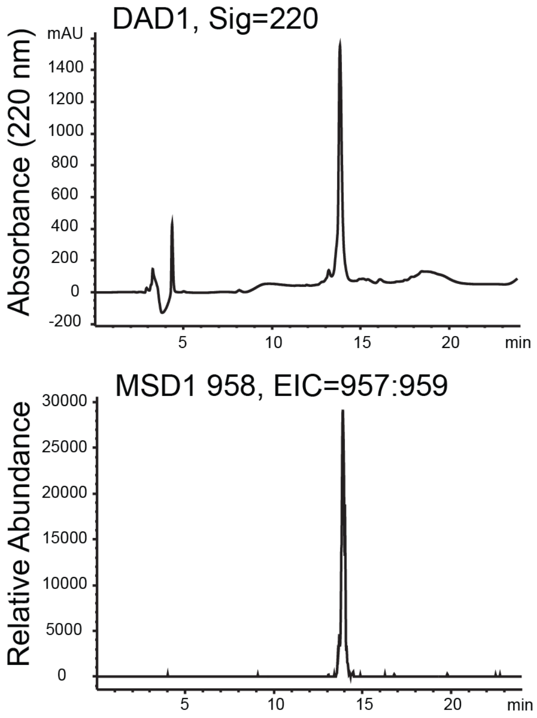

Figure S1. LC/MS Spectra of StIP-TAC peptide PROTAC. Spectra of purified StIP-TAC compound [(Cereblon Ligand 1)-PEG8-*TTY*DFIASGRTGRRNAI]. Absorbance at 220 nm along with extracted ion current (EIC) mass spectrometric detection (MSD) are shown with an expected (M+3)/3 mass = 958.4 (expected mass: 2872.2, found mass = 2871.9).

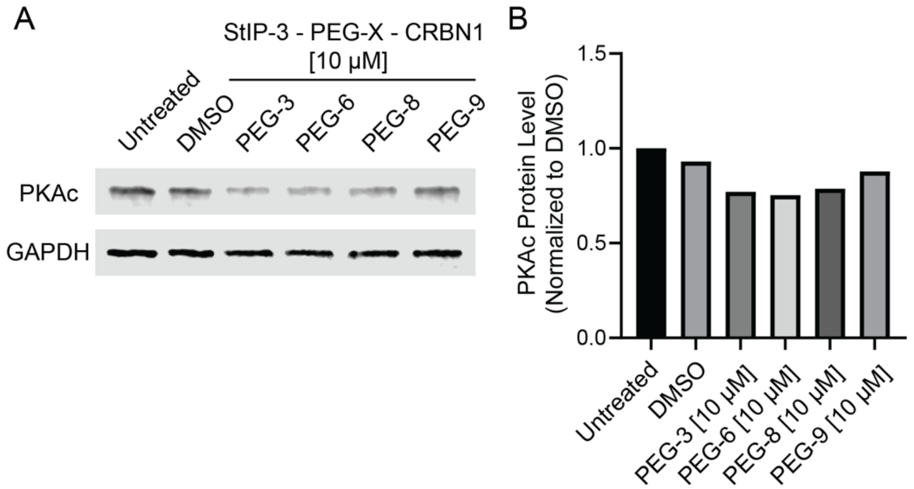

Figure S2. Determination of Linker length for StIP-TAC molecule. Molecules incorporating the StIP-3 parent sequence and Cereblon 1 Ligand (CRBN1) were tested at identical concentrations with PEG linkers of varied lengths (PEG_3, PEG_6, PEG_8, and PEG_9) in MDA-MB-231 cells using SAINT-Protein reagent. Cells were initially serum-starved for 24h then treatments were added alongside SAINT-Protein for 5 hours before harvesting in Laemmli buffer. (A) Representative western blot for cells left untreated, treated with DMSO, or treated with StIP-TAC molecules with varied linker lengths. (B) Densitometric quantification of PKAc protein levels of western blot (n=1) displays a reduction in PKAc protein levels for StIP-TAC molecules incorporating PEG-3, PEG-6, and PEG-8 linkers relative to DMSO control.

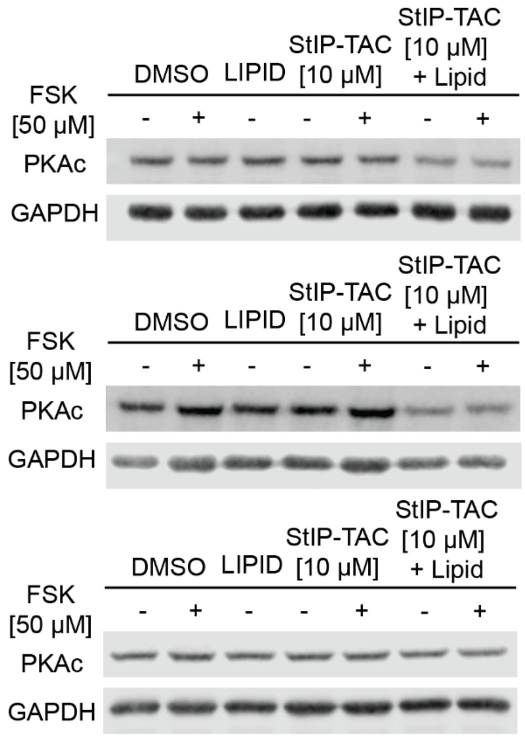

Figure S3: Western blots for PKA degradation. Experiments were performed in triplicate (n=3) in low passage number MDA-MB-231 cells (ATCC HTB-26). Following t = 24 hour starvation in serum-free RPMI media, cells were treated for t = 5 hours with DMSO, SAINT-Protein Lipid reagent alone, 10 μM StIP-TAC, or 10 μM StIP-TAC incubated with SAINT-Protein Lipid reagent per manufacturer instructions. For forskolin-stimulated treatments, cells were incubated for 30 minutes with 50 μM forskolin prior to harvesting in Laemmli sample buffer. Lysates were run via SDS-PAGE and western blotting was performed using mouse anti-PKAc (1:1000, BD Biosciences) and rabbit anti-GAPDH (1:1000, Proteintech).

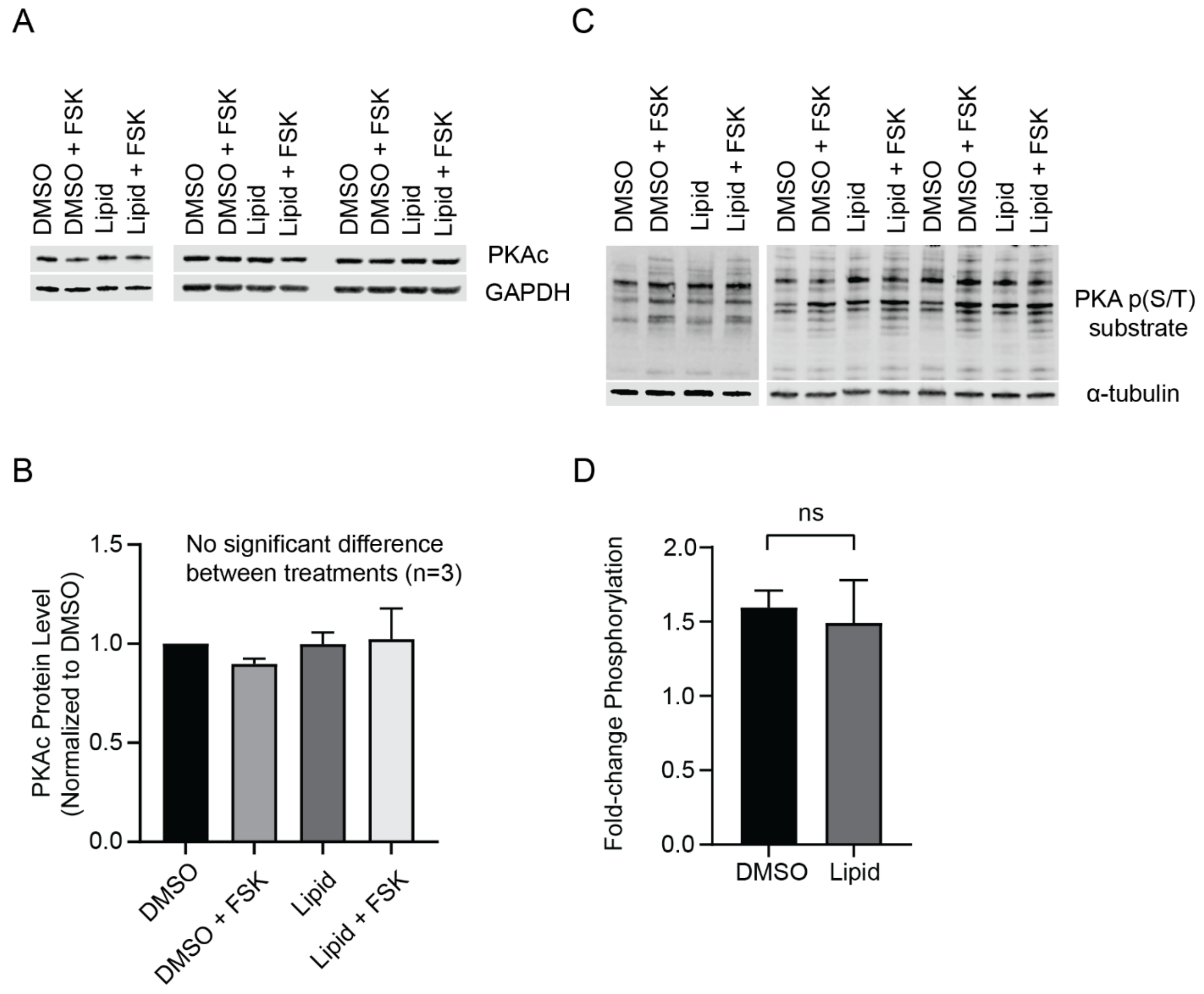

Figure S4. Treatment with SAINT-Protein alone does not influence PKAc protein levels or PKAc substrate phosphorylation. (A) Representative Western blots of cells treated with DMSO or SAINT-Protein (lipid) for 5 hours with or without a 30-minute stimulation with 50 μM forskolin (n=3) and assessed for PKAc protein levels. Cells treated with SAINT-Protein (with or without forskolin stimulation) do not display a reduction in PKAc protein levels relative to DMSO control. (B) Densitometric quantification of Western blots for three independent experiments demonstrates no significant reduction in PKAc protein levels relative to DMSO control for cells treated with SAINT-Protein reagent alone. Quantification was performed via Li-COR Image

Studio. ns, not significant as assessed by one-way ANOVA and Bonferroni's multiple comparisons test. Error bars represent standard deviation. (C) Representative Western blots of cells treated with DMSO or SAINT-Protein (lipid) for 5 hours with or without a 30-minute stimulation with 50 μM forskolin (n=3) and assessed for PKAc p(S/T) substrate protein levels. Cells treated with SAINT-Protein (with or without forskolin stimulation) do not display a reduction in phosphorylated substrate protein levels relative to DMSO control. (D) Densitometric quantification of western blots from three separate experiments demonstrates no statistically significant reduction in substrate phosphorylation for cells treated with SAINT Protein reagent compared to DMSO control. Quantification was performed via Li-COR Image Studio. ns, not significant as assessed by one-way ANOVA and Bonferroni's multiple comparisons test. Error bars represent standard deviation.

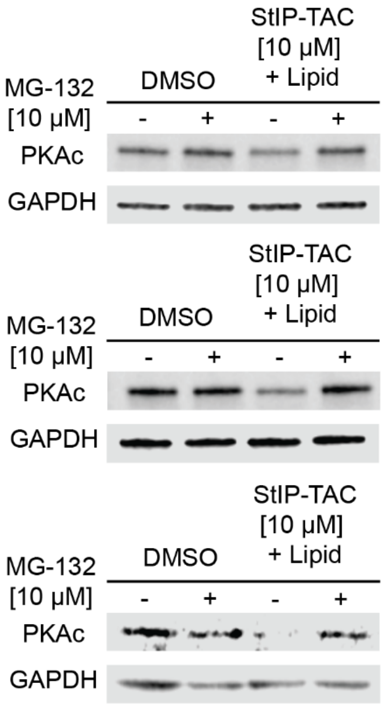

Figure S5: Western blots for MG-132 rescue of degradation. Experiments were performed in triplicate (n = 3) in low passage number MDA-MB-231 cells (ATCC HTB-26). Following t = 24 hour starvation in serum-free RPMI media, cells were treated for t = 5 hours with DMSO or 10 μM StIP-TAC incubated with SAINT-Protein Lipid reagent per manufacturer instructions. For MG-132 treatments, cells were also incubated throughout the 5 hour time course with 10 μM MG-132 prior to harvesting in Laemmli sample buffer. Lysates were run via SDS-PAGE and Western blotting performed using mouse anti-PKAc (1:1000, BD Biosciences) and rabbit anti-GAPDH (1:1000, Proteintech).

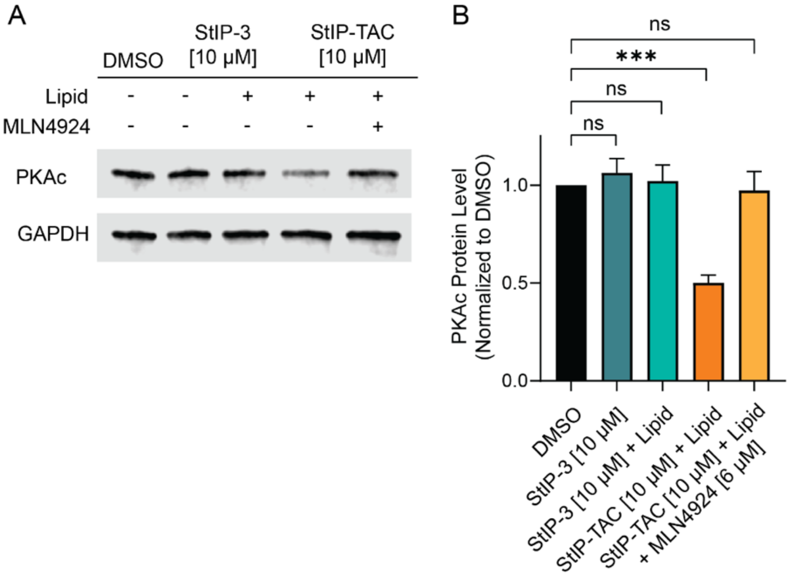

Figure S6: StIP-TAC activity is reliant on the Cereblon ligand and is dependent on activation of the neddylation pathway. (A) Representative Western blot of cells treated for 5 hours with DMSO, the cereblon-unconjugated parental peptide sequence of StIP-TAC (StIP-3) with or without SAINT-Protein lipid reagent, or StIP-TAC with the SAINT-Protein lipid reagent with or without co-treatment with 6 μM of NEDD-activating enzyme (NAE) inhibitor MLN4924. Cell lysates were assessed for PKAc protein levels. Cells treated with StIP-TAC and SAINT-Protein lipid reagent demonstrated a reduction in PKAc levels that was rescued with co-treatment with the NAE inhibitor MLN4924. Cells treated with StIP-3, with or without SAINT-Protein lipid reagent, did not demonstrate a reduction in PKAc levels compared to DMSO. (B) Densitometric quantification of Western blots for three (n = 3) independent experiments demonstrates a significant reduction in PKAc protein levels for treatment with StIP-TAC alongside SAINT-Protein lipid reagent, however, not for the StIP-3 molecule lacking the cereblon ligand, with or without the presence of SAINT-Protein lipid reagent. Co-treatment of cells with the NAE inhibitor MLN4924 rescued PKAc protein levels, indicating the

requirement of activation of the neddylation pathway for StIP-TAC function. Quantification was performed via Li-COR Image Studio. ***$p<0.001$; ns, not significant as assessed by one-way ANOVA and Bonferroni's multiple comparisons test. Error bars represent standard deviation.

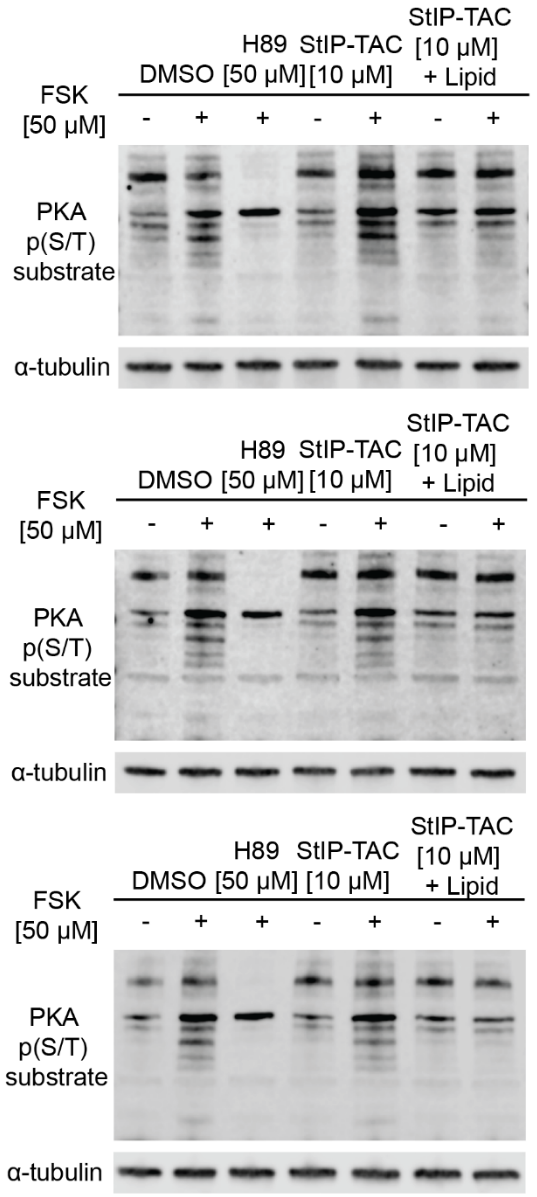

Figure S7: Western blots for PKA substrate phosphorylation. Experiments were performed in triplicate (n=3) in low passage number MDA-MB-231 cells (ATCC HTB-26). Following t = 24 hour starvation in serum-free RPMI media, cells were treated for t = 5 hours with DMSO, SAINT-Protein Lipid reagent alone, 10 μM StIP-TAC, or 10 μM StIP-TAC incubated with SAINT-Protein Lipid reagent per manufacturer instructions. For forskolin-stimulated treatments, cells were incubated for 30 minutes with 50 μM forskolin

prior to harvesting in Laemmli sample buffer. For H89-inhibited treatments, cells were incubated for 30 minutes prior to forskolin stimulation with 50 μM H89. Lysates were run via SDS-PAGE and Western blotting performed anti-phospho serine/threonine PKA substrate (1:1000, Cell Signaling Technology) and anti-tubulin (1:1000, Cell Signaling Technology) primary antibodies.

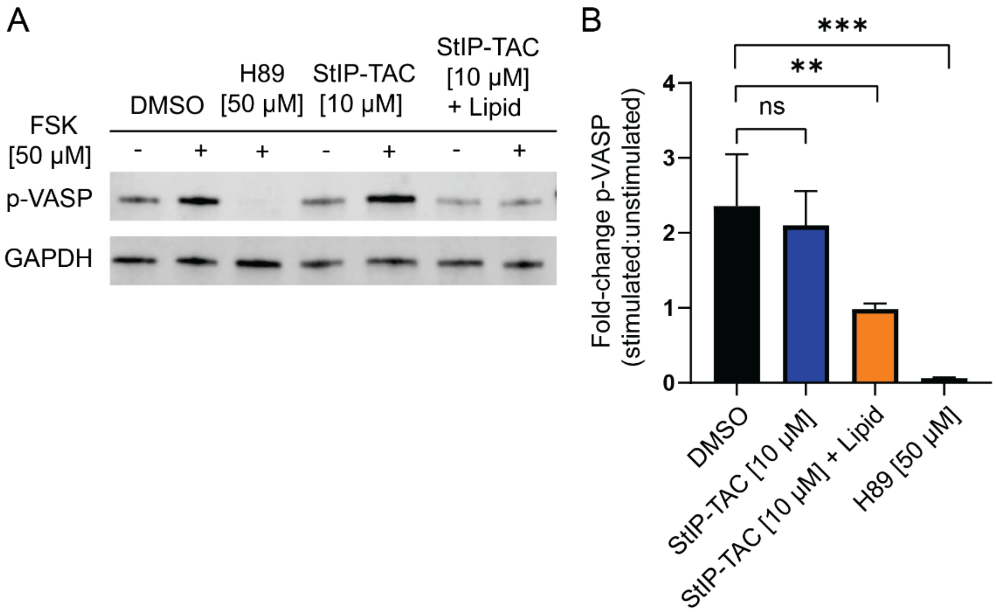

Figure S8: StIP-TAC inhibits phosphorylation of the PKA substrate VASP. VASP = Vasodilator stimulated phosphoprotein. (A) Representative western blot of cells treated with vehicle control, H89, StIP-TAC, or StIP-TAC + SAINT Protein reagent for 5 h with or without 30 min stimulation with 50 μM forskolin (n = 3). Cells treated with H89 and StIP-TAC alongside SAINT protein display a reduction in fold change of phosphorylated VASP levels after forskolin stimulation. (B) Densitometric quantification of Western blots for three independent experiments (n=3) demonstrates a statistically significant reduction in phosphorylated VASP in cells treated with H89 and StIP-TAC with SAINT Protein reagent. Quantification was performed via Li-COR Image Studio. **p=0.010; ***p<0.001; ns, not significant as assessed by one-way ANOVA and Bonferroni's multiple comparisons test. Error bars represent standard deviation.
